# Complete Genome Sequence of *emm*28 Type *Streptococcus pyogenes* MEW123, a Streptomycin-Resistant Derivative of a Clinical Throat Isolate Suitable for Investigation of Pathogenesis

**DOI:** 10.1128/genomeA.00136-16

**Published:** 2016-03-17

**Authors:** Kristin M. Jacob, Theodore Spilker, John J. LiPuma, Suzanne R. Dawid, Michael E. Watson

**Affiliations:** a Division of Pediatric Infectious Diseases, Department of Pediatrics and Communicable Diseases, University of Michigan Medical School, Ann Arbor, Michigan, USA; b Department of Microbiology and Immunology, University of Michigan Medical School, Ann Arbor, Michigan, USA

## Abstract

We present here the complete genome sequence of *Streptococcus pyogenes* type *emm*28 strain MEW123, a streptomycin-resistant derivative of a pediatric throat isolate. The genome length is 1,878,699 bp, with 38.29% G+C% content. The genome sequence adds value to this virulent *emm*28 representative strain and will aid in the investigation of streptococcal pathogenesis.

## GENOME ANNOUNCEMENT

*Streptococcus pyogenes* is a Gram-positive bacterial pathogen responsible for a great diversity of human disease manifestations (1). Genotyping by DNA sequencing of the variable region of the *emm* gene distinguishes *S. pyogenes* isolates; the four most prevalent *emm* types causing pharyngitis and invasive disease in North America are types 1, 3, 12, and 28 (2–4). *S. pyogenes* type *emm*28 has a particular association with female urogenital infections, including vulvovaginitis, endometritis, and puerperal sepsis (5–8). We previously described the *emm*28 strain MEW123 as a streptomycin-resistant derivative of a pediatric throat isolate, which is amenable to genetic manipulation and establishes prolonged carriage in a murine vaginal colonization model (9). We report here the complete MEW123 genome sequence to provide a reference for future studies.

Chromosomal DNA was isolated using the Wizard genomic DNA purification kit (Promega, Madison, WI), and sequenced using the PacBio RS II sequencer (Pacific Biosciences, Menlo Park, CA). Samples were prepared according to the manufacturer’s protocols with the P6 polymerase kit and C4 sequencing reagents, with the exception of an increase to 1 h polymerase binding and 1 h binding to magnetic beads. For library construction, DNA was sheared to fragments of ~23,700 bp and isolated using the BluePippin electrophoresis system (Sage Science, Inc., Beverly, MA). Sequences were collected using one single-molecule real-time (SMRT) cell. This generated 70,159 reads, each with a length of ~16,100 bp, for a total of 1,131.6 Mb of sequence data (~300- to 500-fold coverage). The sequence was assembled using Celera version 8.3rc2 (10, 11). The resulting single scaffold was indexed and aligned against the fastq reads with BWA version 0.7.12, using BWA-MEM. The resulting .SAM file was sorted, indexed, and converted to .bam and .bai files using SAMtools version 1.2 (12, 13). Error correction was performed with Pilon version 1.12 (14) and Harvest tools version 1.2, employing parsnp and gingr (15). Genome overlap at the ends was identified with SeqEdit (DNAStar, Madison, WI), and trimmed manually to have position *dnaA* as the starting point. Preliminary annotation was performed using Prokka version 1.11, with a reference library generated from *emm*28 strain MGAS6180 (accession no. NC_007296.1) (16). Upon submission to GenBank, the annotations were repeated using the NCBI Prokaryotic Genome Annotation Pipeline for database consistency.

The genome sequence contains 1,878,699 bp, with 38.29% G+C% content. The number of predicted coding regions is 1,827, with 18 rRNA and 67 tRNA genes. The PHAge Search Tool (PHAST) identified 1 intact and 2 incomplete prophage regions (17). The Web-based tool CRISPRFinder identified 3 candidate clustered regularly interspaced short palindromic repeat (CRISPR) regions (18). By multilocus sequence typing (MLST), MEW123 was recognized as sequence type 52 (19). MEW123 *emm*28 (cluster E4) has 100% nucleotide identity to *emm*28 of MGAS6180 (8). The MEW123 R28 adhesin gene contained 7 tandem nucleotide repeats of 237 bp each, which is shorter than the MGAS6180 R28 sequence containing 13 repeats (8, 20). Sequence examination identified the pyrogenic exotoxins and superantigens SpeB, SpeC, SpeF, SpeG, SpeJ, and SmeZ. Altogether, these sequence data will benefit future investigations of *S. pyogenes* molecular biology and pathogenesis.

### Nucleotide sequence accession number.

This genome sequence has been deposited in GenBank under the accession no. CP014139. The version described in this paper is the first version.
